# Multi-trait selection for nutritional and physiological quality of cacao genotypes in irrigated and non-irrigated environments

**DOI:** 10.1038/s41598-024-56556-7

**Published:** 2024-03-16

**Authors:** Maurício S. Araújo, Saulo F. S. Chaves, Guilherme R. Pereira, Matheus H. D. Guimarães, Andressa K. S. Alves, Luiz Antônio S. Dias, Carlos A. S. Souza, Marco A. G. Aguilar

**Affiliations:** 1 Department of Agronomy, Federal University of Viçosa, Viçosa, Minas Gerais 36570-900 Brazil; 2 Filogonio Peixoto Experimental Station (ESFIP), Cocoa Research Center, CEPLAC, Augusto Pestana Street, Linhares, Espírito Santo 29990-192 Brazil

**Keywords:** Agricultural genetics, Plant breeding

## Abstract

Water is a scarce, strategic resource and the most important input for economic development, especially in agricultural countries such as Brazil. Cocoa production is directly related to water availability, and, as climate changes, selecting drought-tolerant genotypes is vital to keep cacao crops sustainable. Here, we evaluated cacao genotypes under irrigated and water-stressed conditions and selected drought-tolerant ones based on nutritional and physiological traits. Thirty-nine genotypes were monitored for three years for agronomic traits and higher fruit yield. After this evaluation, the 18 most promising genotypes were evaluated in a randomized block design, under a 2 (with and without irrigation) $\times$ 18 (genotypes) factorial arrangement, with three replicates and five plants per plot. We evaluated seven physiological and 11 nutritional traits, selecting genotypes based on the Genotype-by-Trait Biplot approach. Significant effects (*p* < 0.05) were observed for the nutritional traits N, P, Mg, S, Zn, Cu, Mn and for the physiological traits CO_2_ assimilation rate (A), stomatal conductance (gs), transpiration (E), intercellular and atmospheric CO_2_ concentrations (Ci/Ca), intrinsic water use efficiency (A/gs), instantaneous water use efficiency (A/E), and instantaneous carboxylation efficiency (A/Ci), as determined by analysis of variance. The genotype $\times$ irrigation treatment interaction was significant (*p* < 0.05) for the traits A, gs, and E. Genotypes CP 41, CP 43, and CCN 51 exhibited superior performance for both nutritional and physiological traits (A, gs, and E). In the irrigated environment, CP 41 showed superiority in traits such as P, A/E, A/gs, Mn, S, and Zn. Conversely, under non-irrigated conditions, CP 43 exhibited better performance in nutritional properties, specifically Mn, Mg, and Zn. Notably, in both irrigated and non-irrigated environments, CCN 51 excelled in key physiological traits, including A/Ci, A/E, and A/gs. This robust performance across diverse conditions suggests that these three genotypes possess physiological mechanisms to endure water-stressed conditions. Our research can generate valuable insights into these genotypes informing suitable choices for cocoa cultivation, especially in the context of global climate change.

## Introduction

Among the species of the genus *Theobroma*, cacao (*Theobroma cacao* L.) is the most commercially important. The species is native to the Amazon basin and has great economic, social and environmental importance^1,2^. After fermented, dried, roasted and grounded, its beans are the main raw material of interest for the food and pharmaceutical industries^3–5^ and especially for the production of chocolate^6,7^. South America accounts for 12.5% of the world’s cocoa bean production, and its producing countries earn US$ 2.4 billion/year from its exports. Brazil is the second-largest producer in South America and the seventh-largest producer in the world (269,731 t)^8^. However, there has been a reduction in yield, mainly attributed to the occurrence of witch’s broom disease^9^ and to climate change, particularly due to abiotic and biotic stresses^10,11^. According to the latest report of the Intergovernmental Panel on Climate Change (IPCC), the increase in extreme weather events related to climate change may pose a risk to food security in Brazil due to frequent and extreme droughts^12^. These effects negatively impact cocoa yield^13,14^. According to the World Cocoa Foundation^15^, climate change could cause severe damage to cocoa cultivation in the next decades. This fact is mainly associated with water stress and prolonged periods of drought in the areas of cultivation^16,17^.

Water deficit in cacao was pointed out by Alvim^18^ as the most serious limitation that it can face compared to any other risk, including pests and diseases. Alvim^18^ assured in the 1970s that cocoa yield is considerably more controlled by rainfall than by any other environmental condition. Water scarcity and climate change predicted for the coming decades reinforce the importance of searching for and selecting drought-resistant genotypes with high production capacity. Knowledge in this area is still incipient, given the gravity of the situation: (i) water stress is the main limiting factor for cacao yield^19^; (ii) future scenarios indicate that hotter and drier climates will reduce cacao yield^20^; (iii) there will be a need to expand cultivated areas (which is hardly possible) or select genotypes tolerant to unfavorable climatic conditions^21^. It should be emphasized that, with the growth of cities and increased water demand for human consumption, environmental issues such as licensing and granting of water for cacao cultivation in Brazil should guide discussions on the future of irrigated farming^22^.

From the technological point of view, current research results indicate that cacao can be grown with irrigation in non-traditional areas, such as semi-arid regions^23,24^. Even in traditional growing regions, where water deficit did not exist, complementary irrigation for water supply is becoming necessary, as dry spells are getting more frequent. The southern region of Bahia State, for instance, recorded an extreme dry spell with five months (September/2015 to January/2016) of drought. This dry spell, associated with high temperatures, caused a sharp decrease in cocoa production that year and the following year. Deaths of 13.9% of cacao trees in the region were also recorded, caused by drought or fires^22^.

Cacao originates from the Amazon rainforest and, as such, tolerates a dry season of up to three months^25^. Cacao’s root system concentrates 83% and 86% of thin and thick roots up to a depth of 40 cm, facilitating the cycling of nutrients, but making the cultivation sensitive to long dry spells^16^. Water stress reduces plant development, interfering in several biochemical and physiological processes^26,27^. When cacao is under stress, there is a reduction in leaf area, leading to lower net assimilation rate, stomatal conductance, yield^28^ and transpiration^29^, affecting gas exchange and photosynthesis^30^. Nutrient uptake from the soil and nutrient transportation within the plant are also impaired^31,32^.

Fortunately, the severity of water stress is genotype-dependent, making genetic improvement a viable and sustainable alternative to mitigate its effects by selecting resistant genotypes^33^. Nutritional and physiological traits have been used to select drought-tolerant genotypes. Different selection strategies have been used to evaluate and identify superior genotypes, such as tandem selection, independent levels and selection indices, in addition to those based on principal components^34–37^. Yan and Rajcan^37^ proposed the Genotype-by-Trait (GT) biplot approach to evaluate data of multiple traits, aiming to rank and select the best ones based on joint performance. This approach has already been used in crops such as soybean^37^, common bean^38^, and sunflower^39^. Its use in cocoa has not yet been reported.

The present study aimed to promote the simultaneous selection of genotypes for nutritional and physiological traits under water-stressed conditions using a GT biplot approach. We expect to bring positive impacts on cacao breeding and agriculture by demonstrating how a robust approach such as GT biplot can aid in the selection of drought-tolerant genotypes. The success of this approach will not only contribute to the sustainability of cocoa production but also provide farmers with valuable tools to address specific challenges associated with cacao cultivation.

## Methods

### Genotypes, experiment and design

Eighteen genotypes were evaluated at the “Filogônio Peixoto” Experimental Station (ESFIP) (Table S1), in Linhares (latitude 19° 24′ 52″ S, longitude 40°03′ 54″ W and altitude of 19 m above sea level), Espírito Santo, Brazil. The seeds of these genotypes were pre-germinated, sown in 288 cm$^3$ tubes and irrigated daily by an automated system. After six months, the seedlings were transplanted into polyethylene bags with capacity of 18 L and taken to the greenhouse. The substrate was prepared in a 3:2:1 ratio, resulting from the mixture of soil:sand:cattle manure, and fertilized with 5 kg of single superphosphate and 1 kg of potassium chloride per m$^3$. The trial was set up in a greenhouse in a randomized complete block design, in a 2 $\times$ 18 factorial arrangement (with and without irrigation, and 18 genotypes), with three replicates and five plants per plot, at 12 months of age. In the irrigated treatments, the individuals were kept on a substrate close to field capacity. In the treatments without irrigation, the water supply was gradually suspended. After an acclimatization period, genotypes with more uniform characteristics were divided into two groups, subjected to different treatments related to two distinct irrigation treatments. One group was kept as a control, maintaining constant humidity. Meanwhile, the second group was subjected to a water deficit for a sufficient period to reach and/or fall below − 1.5 MPa in $\Psi _w$ values, indicating a level of “severe water stress” for cacao plants^40^.

It is worth pointing out that the 18 genotypes evaluated were previously selected from the yield monitoring of 39 genotypes under field conditions. Of these 39 genotypes, 11 were selected for their good agronomic traits and higher fruit production per plant, or higher bean yield, quantified during the period of intense drought that occurred between 2014 and 2016 (three years) at the ESFIP. The remaining 7 genotypes were selected at five cacao farms in the municipality of Linhares, adopting the same yield monitoring also carried out during the same three years.

### Evaluation of gas exchange

Gas exchange measurements were made 28 days after suspension of irrigation, on the first fully developed leaf from the apex, between 8:00 and 10:00 a.m., using a portable infrared gas analyzer (IRGA), model LI 6400 (LI-COR, USA), under irradiance of 800 μmol of photons s$^{-1}$. The CO_2_ concentration was 400 μmol m^−2^ s^−1^ (both conditions), and the chamber temperature was 25 $^\circ$C. The following physiological variables were determined, in irrigated and non-irrigated environments: net CO_2_ assimilation rate (A, μmol CO_2_ m$^{-2}$ s$^{-1}$), transpiration (E, mmol H_2_O $\text{m}^{-2} \text{s}^{-1}$), stomatal conductance (gs, mol H_2_O $\text{m}^{-2} \text{s}^{-1}$), intercellular and atmospheric CO_2_ concentration (Ci/Ca, μmol CO_2_ mol air$^{-1}$), intrinsic water use efficiency (A/gs), instantaneous water use efficiency (A/E) and instantaneous carboxylation efficiency (A/Ci).

### Evaluation of macro and micronutrient contents

To determine the contents of macronutrients (N, P, K, Ca, Mg and S) and micronutrients (Fe, Zn, Cu, Mn and B), the same leaves evaluated for gas exchange were collected, also 28 days after suspension of irrigation. Chemical analyses for these determinations were performed according to Malavolta et al.^41^.

### Statistical analysis

#### ANOVAs and Scott–Knott test

Preliminarily, the experimental data were analyzed for homogeneity of variances and normality. Subsequently, the ANOVAs were processed. Once the significance of each effect (Genotype, Irrigation treatment (IT) and Genotype $\times$ IT interaction) for each variable was verified, the means were grouped using the Scott–Knott test^42^ which allowed multiple group comparisons. To avoid multicollinearity in the data set, we excluded variables that originated the derivatives (A, E, and gs) in the multivariate analysis.

The coefficient of determination $R^2$ was determined by:

1 $$\begin{aligned} R^{2} = 1 - \frac{\text {SS}_\text {res}}{\text {SS }_\text {tot}} \end{aligned}$$

where $SS_\text {res}$ is the sum of squares of residuals and $SS_\text {tot}$ is the total sum of squares (proportional to the variance of the nutritional and physiological data).

#### Genotype-by-Trait biplot (GT biplot)

The variables were standardized according to the expression below:

2 $$\begin{aligned} P_{ij}=\frac{T_{ij}-\bar{T_j}\ }{S_j} \end{aligned}$$

where $P_{ij}$ is the standardized value of the genotype *i* for the trait *j*; $T_{ij}$ is the average value of the genotype *i* for the trait *j*; $\bar{T_j}$is the average value of the trait *j* in all genotypes; and S$_j$ is the standard deviation of the trait.

The GT biplot approach was based on the first two principal components (PC) resulting from the singular value decomposition (SVD) of the matrix of standardized variables:

3 $$\begin{aligned} P_{ij}=\left( d\lambda _1^\alpha \xi _{i1}\right) \left( \frac{\lambda _1^{1-\alpha }\tau _{1j}}{d}\right) +\left( d\lambda _2^\alpha \xi _{i2}\right) \left( \frac{\lambda _2^{1-\alpha }\tau _{2j}}{d}\right) +\varepsilon _{ij} \end{aligned}$$

where $\xi _{i1}$ and $\xi _{i2}$ are the eigenvalues for PC1 and PC2, respectively, for the genotype *i*; $\tau _{1j}$ and $\tau _{2j}$ are the eigenvalues for PC1 and PC2, respectively, for the trait *j*, and $\varepsilon _{ij}$ is the adjusted residual of PC1 and PC2 for the genotype *i* in the trait *j*; $\lambda _1$ and $\lambda _2$ are the eigenvalues for PC1 and PC2, respectively, and $\alpha$ is the partition factor of the singular value. If $\alpha$ = 1, the biplot is said to be genotype-focused and is suitable for comparing genotypes. When $\alpha = 0$, the biplot is said to be trait-focused and is suitable for viewing correlations between traits. The GT biplot was constructed by plotting $(d\lambda _1^{\ \alpha }\xi _{i1})$ against $(d\lambda _2^{\ \alpha }\xi _{i2})$, for genotypes, and plotting ${[(\lambda }_1^{1-\alpha }\tau _{1j})/d]$ against ${[(\lambda }_2^{1-\alpha }\tau _{2j}/d)]$, for trait in the same plot. The analysis described was performed using the traits’ mean values between irrigated and non-irrigated treatments. The heatmap for Pearson’s correlation was created using the ComplexHeatmap package^43^. The GT biplot approach was processed using the metan package^44^, both in R software^45^.

### Ethical statement

The plant species used here is a cultivated plant (*T. cocoa* L.), and the genotypes employed were provided by the “Filogônio Peixoto” Experimental Station (ESFIP), located in the state of Espírito Santo, Brazil. We confirm that we have complied with all the necessary regulations for this type of research.

## Results

### Effects of genotype, irrigation, and their interaction on nutritional and physiological traits of cocoa

The effect of genotypes (G) was significant (*p* < 0.05) for the nutritional variables N, P, Mg, S, Zn, Cu and Mn and physiological variables A, gs, E, Ci/Ca, A/gs, A/E and A/Ci. The irrigation treatment (IT) effect was significant for the nutritional variables P, K, Ca, Mg, S, Fe, Mn and B and the physiological variables A, gs, E and Ci/Ca. The G $\times$ IT interaction was significant for the physiological variables A, gs and E (Table 1).

**Table 1:** ANOVA for nitrogen (N), phosphorus (P), potassium (K), calcium (Ca), magnesium (Mg), sulfur (S), iron (Fe), zinc (Zn), copper (Cu), manganese (Mn), boron (B), net CO_2_ assimilation rate (A), stomatal conductance (gs), transpiration (E), internal carbon concentration (Ci/Ca), intrinsic water use efficiency (A/gs), instantaneous water use efficiency (A/E), and instantaneous carboxylation efficiency (A/Ci) in 18 cocoa genotypes evaluated in irrigated and non-irrigated environments.

Category	Traits	Mean squares					Mean	CV (%)	R^2^
		Block	Genotype (G)	Irrigation treatment (IT)	G x IT	Residual			
Nutritional	N	5.72	8.59*	0.26	3.87	4.32	25.93	8.02	0.43
	P	0.7	0.30*	4.46*	0.08	0.1	2.46	12.86	0.63
	K	0.52	5.07	26.81*	2.99	5.22	14.93	15.3	0.31
	Ca	165.57	6.07	66.40*	6.55	6.09	12.27	20.11	0.59
	Mg	0.32	1.04*	12.44*	0.59	0.53	4.48	15.25	0.52
	S	0.74	0.19*	1.99*	0.07	0.11	2.58	12.88	0.52
	Fe	10700.93	473.46	67800.33*	355.04	365.71	104.87	18.24	0.81
	Zn	70.56	205.14*	118.23	127.58	78.34	47.27	18.72	0.52
	Cu	4.75	3.33*	1.33	0.59	0.78	4.33	20.38	0.59
	Mn	176178.5	100707.31*	1191120.04*	55945.19	47530.67	4.33	20.38	0.56
	B	31.84	45.62	2690.01*	79.13	56.84	39.62	19.03	0.55
Physiological	A	2.6	2.72*	33.93*	2.23*	0.75	3.69	23.45	0.70
	gs	0.0004	0.0006*	0.0191*	0.0011*	0.0003	0.05	38.34	0.68
	E	0.26	0.39*	4.58*	0.55*	0.2	1.26	35.19	0.61
	Ci/Ca	646.9	117.07*	926.81*	50.62	47.55	20.78	33.18	0.61
	A/gs	3794.4	3800.74*	961.21	967.56	953.77	88.88	34.74	0.57
	A/E	0.03	5.45*	0.04	0.8	1.08	3.27	31.97	0.59
	A/Ci^1^	0	0*	0	0	0	0.02	0	0.62

Coefficient of variation (CV) and coefficient of determination (R^2^). The degrees of freedom for block, genotypes, irrigation treatment, G $\times$ IT, and Residual are 2, 17, 1, 17, and 70, respectively. *Significant at 5% probability level by the F test. ^1^Showed mean square values below one and with many decimal places and was then represented by zeros.

### Traits’difference between cacao genotypes under different irrigation treatments

The genotypes had different responses for the physiological traits evaluated, allowing the selection of superior individuals. CCN 51, CP 196, CP 223 and CP 236 showed predictable behavior in both environments evaluated, indicating tolerance to water stress (Table 2).

**Table 2:** Scott–Knott test for grouping of means (*p* < 0.05) relating cocoa genotypes in irrigated (IR) and non-irrigated (NIR) irrigation treatments for the physiological variables A, gs, and E.

Genotypes	Variables*					
	${A^{1}}$		*gs*		*E*	
	IR	NIR	IR	NIR	IR	NIR
BN 34	3.28bA	2.47bB	0.05bA	0.03aA	1.18bA	1.08aA
CCN 51	5.89aA	4.67aA	0.04bA	0.03aA	0.80bA	0.99aA
CEPEC 2002	3.87bA	2.59bB	0.06bA	0.03aB	1.39aA	1.03aA
CP 176	4.00bA	2.67bA	0.05bA	0.03aA	1.02bA	1.13aA
CP 196	4.59aA	3.15bA	0.05bA	0.04aA	1.18bA	1.31aA
CP 197	3.37bA	2.53bA	0.04bA	0.02aB	0.95bA	0.57aA
CP 223	2.14bA	1.83bA	0.05bA	0.04aA	1.06bA	1.21aA
CP 234	4.83aA	3.35aB	0.10aA	0.02aB	2.43aA	0.76aB
CP 236	2.84bA	2.16bA	0.08aA	0.04aA	2.11aA	1.16aA
CP 41	3.22bA	2.36bA	0.03bB	0.07aA	0.78bB	1.54aA
CP 43	5.62aA	4.26aB	0.05bA	0.03aB	1.33aA	1.00aA
CP 49	4.87aA	3.97aB	0.07aA	0.03aB	1.62aA	0.89aB
ESFIP 02	4.86aA	3.58aA	0.06aA	0.03aB	1.49aA	0.97aA
ESFIP 04	5.04aA	4.26aB	0.05bA	0.04aA	1.37aA	1.12aA
PH 16	4.91aA	4.14aB	0.08aA	0.05aA	1.82aA	1.33aA
PS 1319	4.32aA	2.69bA	0.08aA	0.03aB	1.47aA	0.81aB
SJ 02	4.59aA	2.87bB	0.11aA	0.02aB	2.36aA	0.82aB
TSH 1188	4.33aA	2.85bA	0.09aA	0.04aB	2.04aA	1.26aB
Means	4.25	3.13	0.06	0.04	1.47	1.05

*Means followed by the same lowercase letters in the columns and uppercase letters in the rows compare genotypes and irrigation treatments, respectively, by the Scott–Knott test, at 5% probability level. ^1^See codes in Table 1.

The 18 genotypes were divided into two groups related to the contents of the nutrients N, P, Mg, S, Mn, Cu and Zn (Table 3). These contents ranged from 24.2 to 27.5 (N), 2.1 to 2.9 (P), 4.0 to 5.6 (Mg), 2.3 to 2.9 (S), 459.6 to 986.0 (Mn), 3.3 to 5.8 (Cu) and 37.6 to 56.3 (Zn) under the evaluated conditions. Overall, the genotypes ESFIP 04, CP 223, CP 43 and PH 16 had higher nutrient contents under the evaluated conditions. Furthermore, the nutrient content was generally higher in the irrigated environment than in the water-stressed environment (Table 4).

**Table 3:** Scott–Knott test for grouping of means comparing the contents of the nutrients N, P, K, Mg, S, Fe, Zn, Cu, Mn, and B in 18 cocoa genotypes. ^1^See codes in Table 1. Means followed by the same letter do not differ significantly from each other, at 5% probability level.

**Genotype**	N^1^		P		K		Mg		S		Fe		Zn		Cu		Mn		B	
	IR	NIR	IR	NIR	IR	NIR	IR	NIR	IR	NIR	IR	NIR	IR	NIR	IR	NIR	IR	NIR	IR	NIR
ESFIP 04	28.47a	27.63a	3.23a	2.71a	16.25a	17.29a	5.88a	6.13a	2.83a	3.04a	156.67a	103.67a	67.00a	60.00a	5.67a	6.00a	1158.33a	813.67a	57.00a	50.33a
CP 234	28.40a	27.23a	3.04a	2.65a	15.84a	16.67a	5.35a	5.67a	2.79a	3.04a	155.00a	89.00a	57.00a	58.33a	5.67a	5.67a	1122.00a	812.00a	50.33a	37.67a
CCN 51	27.81a	26.90a	3.00a	2.50a	15.83a	16.67a	4.84a	5.40a	2.73a	2.97a	142.33a	87.67a	53.67a	55.67a	5.67a	5.00a	1074.00a	793.67a	49.67a	35.67a
CP 223	27.56a	26.86a	2.94a	2.45a	15.83a	16.46a	4.79a	5.38a	2.69a	2.83a	140.67a	87.67a	53.33a	55.67a	5.33a	5.00a	887.33b	673.00a	48.00a	35.33a
PH 16	27.28a	26.83a	2.79a	2.45a	15.83a	15.84a	4.71a	5.35a	2.64a	2.82a	137.00a	86.33a	50.00a	51.67a	5.00a	4.67a	839.67b	660.33a	48.00a	35.00a
ESFIP 02	27.02a	26.76a	2.76a	2.31a	15.63a	15.63a	4.71a	5.32a	2.64a	2.80a	133.67a	85.33a	48.67a	51.00a	5.00a	4.33b	805.33b	621.00a	45.33a	34.67a
CEPEC 2002	26.60a	26.60a	2.68b	2.29a	15.42a	15.63a	4.65a	5.29a	2.51a	2.77a	132.67a	82.67a	48.67a	51.00a	4.67a	4.33b	803.33b	610.33a	45.33a	34.33a
PS 1319	26.53a	26.44a	2.63b	2.29a	15.00a	15.63a	4.54a	5.19a	2.47a	2.74a	132.33a	80.67a	48.67a	47.67a	4.67a	4.33b	778.33b	581.67a	45.00a	34.00a
TSH 1188	26.51a	25.88a	2.62b	2.27a	14.58a	15.42a	4.52a	5.17a	2.44a	2.70a	131.00a	80.00a	48.33a	47.33a	4.33b	4.33b	760.33b	567.00a	45.00a	33.67a
CP 49	26.41a	25.78a	2.59b	2.27a	14.17a	15.21a	4.44a	5.15a	2.37a	2.70a	130.67a	79.67a	43.33b	47.33a	4.33b	4.00b	757.67b	556.67a	44.67a	33.33a
SJ 02	25.34b	25.67a	2.59b	2.21a	14.17a	15.21a	4.29a	5.02a	2.37a	2.63a	129.00a	76.33a	42.67b	46.67a	4.33b	4.00b	757.67b	550.00a	43.33a	33.33a
BN 34	25.32b	25.34a	2.58b	2.16a	13.96a	15.21a	4.27a	5.02a	2.36a	2.60a	127.67a	76.00a	42.33b	44.67a	4.00b	3.67b	671.67b	545.00a	42.67a	33.00a
CP 236	24.92b	25.20a	2.54b	2.12a	13.96a	15.00a	4.00a	4.92a	2.23a	2.59a	123.00a	75.33a	41.00b	44.00a	3.67b	3.67b	644.33b	537.00a	42.00a	32.67a
CP 41	24.59b	25.11a	2.52b	2.12a	13.75a	14.38a	4.00a	4.82a	2.20a	2.57a	115.67a	74.33a	40.67b	44.00a	3.67b	3.67b	641.00b	482.00a	41.67a	32.67a
CP 197	24.59b	25.04a	2.40b	2.09a	12.92a	14.38a	3.92a	4.79a	2.19a	2.55a	114.67a	74.00a	39.67b	43.67a	3.67b	3.67b	640.00b	463.00a	41.00a	32.33a
CP 176	24.18b	24.57a	2.37b	2.08a	12.29a	14.38a	3.83a	4.65a	2.19a	2.51a	114.67a	68.67a	39.67b	43.33a	3.67b	3.33b	615.00b	415.00a	38.67a	32.33a
CP 43	24.15b	24.22a	2.34b	1.90a	12.29a	14.38a	3.63a	4.44a	2.16a	2.51a	111.33a	67.00a	37.33b	42.00a	3.33b	3.33b	590.33b	341.67a	38.33a	32.33a
CP 196	21.96b	23.82a	2.29b	1.74a	12.09a	14.38a	3.46a	4.36a	2.09a	2.44a	110.67a	62.33a	30.00b	35.67a	3.33b	3.00b	577.67b	320.33a	37.00a	30.67a

**Table 4:** Scott–Knott test for grouping of means (p< 0.05) comparing the contents of the nutrients P, K, Mg, S, Fe, Zn, Cu, and B in irrigated and non-irrigated environments. ^1^See codes in Table 1. Means followed by the same letter in the column do not differ significantly from each other, at 5% probability level.

**Environment**	Contents of nutrients									
	N^1^	P	K	Mg	S	Fe	Zn	Cu	Mn	B
Irrigated	25.98a	2.66a	15.43a	5.11a	2.71a	129.93a	48.31a	4.44a	784.67a	44.61a
Non-irrigated	25.88a	2.26b	14.43b	4.44b	2.44b	79.81b	46.22a	4.22a	574.63b	34.63b
Means	25.93	2.46	14.93	4.78	2.58	104.87	47.27	4.33	679.65	39.62

**Figure 1:**
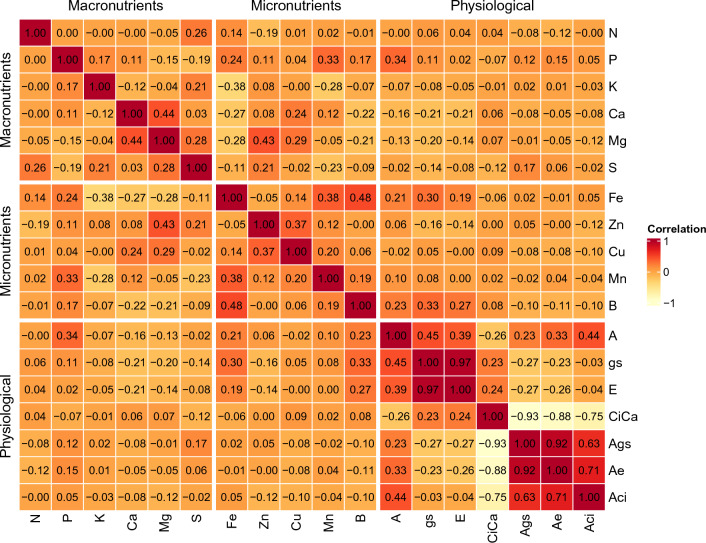
Pearson’s correlation coefficients between nutritional and physiological traits evaluated in 18 cocoa genotypes, in irrigated and non-irrigated environments. See codes in Table 1 for physiological and nutritional traits.

Most correlations between traits were low, except for physiological traits. The highest correlation coefficients were observed between Ci/Ca and A/gs (r=-0.93), A/gs and A/E (r=0.92), A/E and A/Ci (r=0.71), A/gs and A/Ci (r=0.63), Fe and B (r=0.48), and Zn and Mg (r=0.43) (Fig. 1).

### Selection of cacao genotypes based on the nutritional and physiological traits

**Figure 2:**
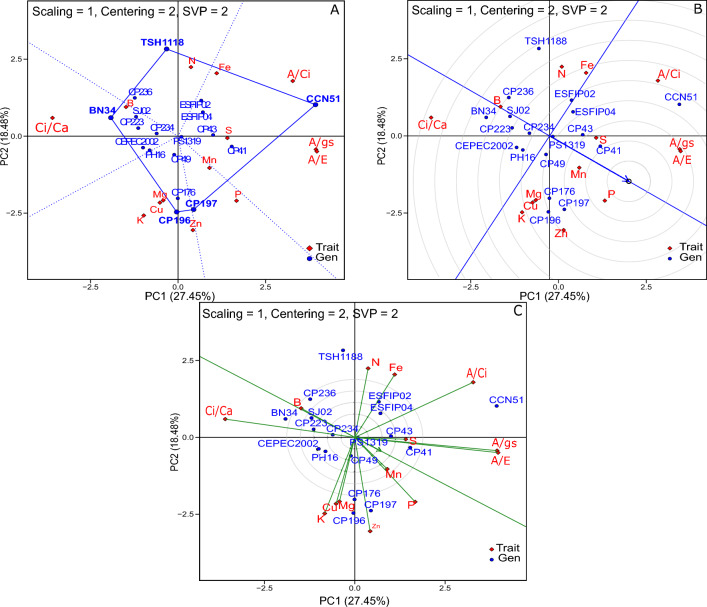
GT Biplot approach in the evaluation of the performance of 18 cocoa genotypes in irrigated environment: (**A**) “Which-Won-Where/What”, (**B**) ranking of genotypes, (**C**) discriminant versus representative. PC 1 = Principal component 1, PC 2 = Principal component 2. ^1^See codes in Tables 1 and 2 for physiological and nutritional traits.

Three plots were generated using the GT biplot method to understand the relationship between genotypes and nutritional and physiological traits (Fig. 2). The“Which-Won-Where/What” biplot was used to identify the superior genotypes for a set of traits in the irrigated environment. At the vertices of the polygon lies the superior genotype in its respective sector (Fig. 2A). The polygon was divided into five sectors, and the superior genotypes for each sector are TSH 1188 in Sector 01, CCN 51 in Sector 02, CP 197 in Sector 03, CP 196 in Sector 04, and BN 34 in Sector 05.

The “ideal”genotypes are those whose projections of the ATC (Average tester coordination) on the horizontal axis correspond to the largest vectors, that is, those genotypes that have combined high performance and good stability for the different traits. CP 41, CP 43, CP 197 and CP 176 were the “ideal”genotypes for most of the traits (Fig. 2B). Nevertheless, CCN 51 had a superior performance in A/Ci, A/gs and A/E. Traits with longer vectors and with smaller angles in the ATC in the biplot C are considered more discriminant and representative, respectively (Fig. 2C). In general, the traits were able to discriminate the genotypes well, especially A/Ci, A/gs, A/E, Ci/Ca, K and Zn. S, A/gs and A/E were the most representative traits.

**Figure 3:**
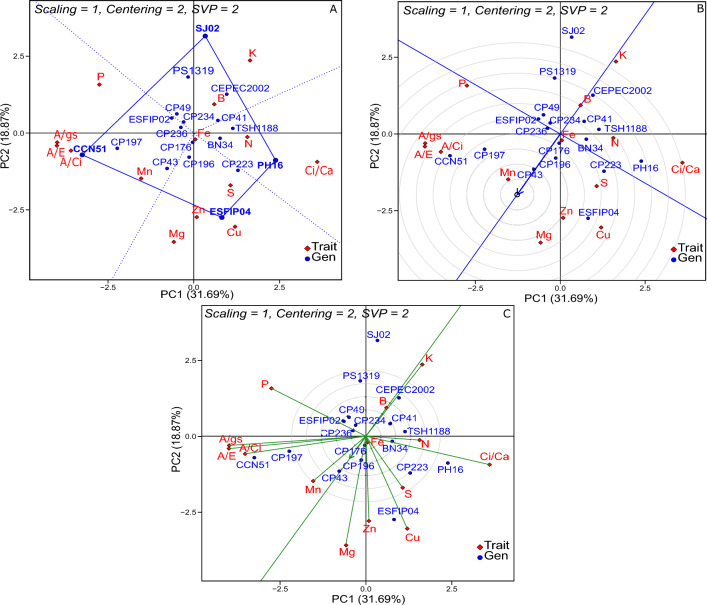
GT Biplot approach in the evaluation of the performance of 18 cocoa genotypes in non-irrigated environment: (**A**) “Which-Won-Where/What”, (**B**) ranking of genotypes, C) discriminant versus representative. PC 1 = Principal component 1, PC 2 = Principal component 2. ^1^See codes in Tables 1 and 2 for physiological and nutritional traits.

In the non-irrigated environment, the genotypes with the best performance were SJ-02 (sector 1), PH 16 (sector 2), ESFIP 04 (sector 3) and CCN 51 (sector 4) (Fig. 3A). The last-mentioned genotype showed similar behavior to that observed in the irrigated environment, mainly with high performance in the respective sector for the traits A/Ci, A/gs and A/E. The genotypes CP 43, CP 196, CP 197 and CP 176 were superior in the non-irrigated environment (Fig. 3B). CP 196 and CP 197 showed predictable performance in both environments. The traits Ci/Ca, Cu, Mg, K, P, A/gs and A/E were the most discriminant, while S, Ci/Ca, A/gs, and A/E were the most representative (Fig. 3C).

## Discussion

Cacao has wide genetic diversity for agronomic^46^ and biochemical^47^ traits. The difference in the concentration of nutrients in cacao is due to differences in the genotypic constitution, requirement of nutrients and efficiency in their use. Furthermore, cacao has several adaptive mechanisms acting in favor of the survival and development of the plant under water stress, which are also genotypic-dependent^48^. It is expected that a drought-tolerant genotype reunites several physiological resources that enable it to thrive in yield and biomass production, even in limited environments. Following this principle and leveraging the genetic differences in physiological behavior and nutrient uptake under water-stressed conditions, we selected genotypes that had more evidence of being drought-tolerant.

When cacao faces water stress, significant changes occur in parameters such as leaf area, thickness, and leaf number^49^ The initial stage of seed development is dramatically impacted^48^. These alterations result in constraints on sugar transport and lipid availability in the seeds. As a common adaptive response to this challenging scenario, cacao tends to reduce biomass allocation to the roots. This is manifested in the frequent occurrence of premature leaf abscission, an effective strategy to minimize water loss through transpiration. Additionally, there is an observed development of thicker leaves, accompanied by a reduction in the number of stomata on the leaves^50,51^. These adaptations highlight the complex physiological response of cacao to water stress, underscoring specific mechanisms that contribute to its survival in under conditions. Nitrogen, in the form of nitrate in the vacuole, confers greater tolerance to water stress by contributing to the maintenance of cell turgor. Phosphorus and potassium, when accumulated in the plant, improve osmotic adjustment. Changes in phosphorus concentration may lead to higher efficiency in water use and stomatal conductance^3^. The absorption of magnesium, sulphur and manganese is also influenced by water stress. Deficiency of these macronutrients and micronutrients can directly affect photosynthesis^52^. This complex interaction between soil nutrients and the physiological responses of cacao to water stress allows us to comprehend the mechanisms of adaptation and tolerance of plants under water stress conditions.

In this study, the CP 43 genotype had better performance for nutritional and physiological traits in both irrigation treatments. However, CCN 51 showed a better response for tolerance to water stress. Despite the scarcity of studies on the physiological and biochemical response of these genotypes, it is noteworthy that the Ecuadorian clone CCN 51 displays high rates of CO_2_ assimilation (A) under open cultivation conditions compared to shaded agroforestry systems. When subjected to high radiation, CCN 51 tends to increase non-photochemical quenching of Chlorophyll *a*^53^. Furthermore, CCN 51 maintains high stomatal conductance rates (between 250-350 mmol m^-2^ s^−1^)^54^, exhibiting water use efficiency around 2.3 mmol mol^−1^. In physiological response to water stress, it is highlighted that CCN 51 shows low cadmium (Cd) absorption^55^, which is significant as elevated levels of this mineral can trigger competition with other essential nutrients such as Zn, Mn, Fe, and Cu^56^. These characteristics reinforce the adaptability and resilience of CCN 51 to adverse water stress conditions. Thus, while the CP 43 genotype showed superior performance in certain aspects, such as nutritional characteristics, it is CCN 51 that stands out in terms of water efficiency and stress tolerance, showcasing physiological adaptations that favor its performance under specific cultivation conditions^57^. These findings underscore the importance of considering not only overall performance but also specific responses to environmental factors when evaluating the suitability of different genotypes for specific agricultural practices.

It is worth remembering that cacao has photosynthetic characteristics of a shade-tolerant species, with a net carbon assimilation rate (A) at radiation levels ranging from 200 to 750 μmoL ${\text{m}}^{-2} {\text{s}}^{-1}$, with light compensation point ranging from 5 to 57 μmoL ${\text{m}}^{-2} {\text{s}}^{-1}$, and maximum value of A ranging from 1 to 8 μmoL ${\text{m}}^{-2} {\text{s}}^{-1}$. For this reason, most cacao plantations are carried out in shaded environments^58,59^. Soil water deficit reduces cocoa production, decreases seed size, and influences leaf renewal and flowering^18^. According to Carr and Lockwoods^60^, premature leaf fall, yellowing of basal leaves, wilting, small leaves and reduced growth are visible symptoms caused by drought in cocoa trees.

The physiological variables A, gs, Ci/Ca and E had higher values in the irrigated environment. The reduction in the Ci/Ca rate may be associated with the reduction in the stomatal conductance rate (gs) of the plants^61^. As shown in this study, there are cacao genotypes with alleles for drought tolerance, and the species has wide genetic variability. Therefore, it is possible and desirable to create breeding programs aimed at resistance to water stress. It is also worth remembering that water is a scarce and finite resource, which needs to be rationally used. In conclusion, genotypes CP 41, CP 43 and CCN 51 showed promise in terms of tolerance to water stress and accumulation of nutrients. In the comparative analysis between genotypes, it was observed that CP 41 showed superiority in various characteristics, specifically P, A/E, A/gs, Mn, and S when cultivated under irrigated conditions. Conversely, under non-irrigated conditions, CP 43 exhibited better performance in the nutritional features Mn, Mg, and Zn. Notably, the genotype CCN 51 excelled in key physiological traits, A/Ci, A/gs, and A/E, in both environments, indicating remarkable tolerance to adverse environmental conditions. As a result, this study provides an in situ assessment of these cacao genotypes, followed by a simultaneous selection of physiological and nutritional traits through the GT biplot approach. Grounded in multivariate analysis, this method identifies the strengths and weaknesses of each genotype, enhancing the selection process with greater robustness and efficiency. The obtained results have the potential to offer valuable insights into these genotypes, aiding informed decision-making for cultivation on farms or in large production areas.

### Supplementary Information

Supplementary Table S1.

## Data Availability

The dataset used and/or analysed during the current study is available from the corresponding author upon reasonable request.
