# Supplementary material for: Multi-trait selection for nutritional and physiological quality of cacao genotypes in irrigated and non-irrigated environments
Source: Sci Rep. 2024 Mar 16;14:6368. doi: 10.1038/s41598-024-56556-7 (PMC11344134; doi:10.1038/s41598-024-56556-7)
Supplement: Supplementary file 1 — Supplementary Table S1. [file 41598_2024_56556_MOESM1_ESM.pdf]

Multi-trait selection for nutritional and physiological quality of cacao genotypes in irrigated and non-irrigated environments

January 11, 2024

Table S1: Description of the 18 cocoa clones in terms of genetic and geographical origin.

Genotypes	Genetic origin	Geographical origin
BN 34	Primary germplasm*	Brazil
CCN 51	(ICS-95 x IMC-67) x CCN-1	Ecuador
CEPEC 2022	Primary germplasm*	Ecuador
CP 41	Primary germplasm*	Brazil
CP 43	Primary germplasm*	Brazil
CP 49	TSA-644 x CCN-51	Brazil
CP 196	Primary germplasm*	Brazil
CP 197	CCN-51 x TSH-1188	Brazil
CP 176	TSA-644 x CCN-51	Brazil
CP 223	Primary germplasm*	Brazil
CP 234	Primary germplasm*	Brazil
CP 236	Primary germplasm*	Brazil
ESFIP 02	Primary germplasm*	Brazil
ESFIP 04	Primary germplasm*	Brazil
PH 16	Primary germplasm*	Brazil
PS 1319	Primary germplasm*	Brazil
SJ 02	Primary germplasm*	Brazil
TSH 1188	Primary germplasm*	Brazil

* These genotypes originated from selections on farms or at the center of diversity.
